# Diffusion Boundary Layers Ameliorate the Negative Effects of Ocean Acidification on the Temperate Coralline Macroalga *Arthrocardia corymbosa*

**DOI:** 10.1371/journal.pone.0097235

**Published:** 2014-05-13

**Authors:** Christopher E. Cornwall, Philip W. Boyd, Christina M. McGraw, Christopher D. Hepburn, Conrad A. Pilditch, Jaz N. Morris, Abigail M. Smith, Catriona L. Hurd

**Affiliations:** 1 Department of Botany, University of Otago, Dunedin, New Zealand; 2 National Institute for Water and Atmospheric research (NIWA) Centre of Physical and Chemical Oceanography, Dunedin, New Zealand; 3 School of Chemistry and Biochemistry, Clark University, Worcester, Massachusetts, United States of America; 4 School of Science and Technology, University of New England, Armidale, Australia; 5 Department of Biological Sciences, University of Waikato, Hamilton, New Zealand; 6 Institute for Marine and Antarctic Studies, University of Tasmania, Hobart, Tasmania, Australia; University of California- Santa Barbara, United States of America

## Abstract

Anthropogenically-modulated reductions in pH, termed ocean acidification, could pose a major threat to the physiological performance, stocks, and biodiversity of calcifiers and may devalue their ecosystem services. Recent debate has focussed on the need to develop approaches to arrest the potential negative impacts of ocean acidification on ecosystems dominated by calcareous organisms. In this study, we demonstrate the role of a discrete (i.e. diffusion) boundary layer (DBL), formed at the surface of some calcifying species under slow flows, in buffering them from the corrosive effects of low pH seawater. The coralline macroalga *Arthrocardia corymbosa* was grown in a multifactorial experiment with two mean pH levels (8.05 ‘ambient’ and 7.65 a worst case ‘ocean acidification’ scenario projected for 2100), each with two levels of seawater flow (fast and slow, i.e. DBL thin or thick). Coralline algae grown under slow flows with thick DBLs (i.e., unstirred with regular replenishment of seawater to their surface) maintained net growth and calcification at pH 7.65 whereas those in higher flows with thin DBLs had net dissolution. Growth under ambient seawater pH (8.05) was not significantly different in thin and thick DBL treatments. No other measured diagnostic (recruit sizes and numbers, photosynthetic metrics, %C, %N, %MgCO_3_) responded to the effects of reduced seawater pH. Thus, flow conditions that promote the formation of thick DBLs, may enhance the subsistence of calcifiers by creating localised hydrodynamic conditions where metabolic activity ameliorates the negative impacts of ocean acidification.

## Introduction

The uptake of a large proportion of anthropogenic CO_2_ emissions is currently reducing surface seawater pH (a process termed ocean acidification, OA), and is projected to decrease pH by approximately 0.35 units by 2100 [1]–[3]. Perturbation experiments simulating OA reveal that many calcifying organisms experience lower net calcification rates, resulting in considerably reduced net calcification [4], [5]. Other detrimental physiological and ecological effects include altered behavioural traits, and reduced reproduction and overall survival for some species [6]–[8]. Together, these detrimental effects may fundamentally change the structure and function of ecosystems dominated by calcareous organisms, resulting in changes in their productivity, biodiversity and stability [9]–[11]. Cold temperate coastal ecosystems may be particularly under threat as calcifying organisms here straddle multiple trophic levels and also deliver essential ecosystem services [12], [13]. For example, coralline algae are calcifying primary producers that cover large areas of temperate rocky shores beneath kelp canopies [14], [15], provide settlement cues for invertebrate larvae [16]–[18], and are pivotal to the functioning of these ecosystems [19]. Little is known about the cumulative effects of OA on such potentially vulnerable temperate ecosystems, or on what type of ecosystem(s) might replace them over large expanses of coralline-dominated coastline.

There has been recent discussion on the ability of photosynthetic calcifying organisms to biologically modify their local pH environment, thereby ameliorating the negative effects of ocean acidification [20]–[26]. However, this hypothesis remains untested by long-term perturbation experiments. In addition, discussion of the effects of climate change on coastal biota [27] has so far included little mention of the important role that organism-seawater flow interactions (ecomechanics)[28] can play in modifying the effects of OA, particularly under slow water flows. Recent studies suggest that OA could be less detrimental to calcifying species that photosynthesize, such as coralline algae, growing in slow-flow compared to fast-flow habitats. This has been suggested because the diffusion boundary layer (hereafter ‘DBL’) provides a thin but significant biologically-controlled buffer between the calcifying organism's external structure and the outer bulk seawater, potentially reducing rates of dissolution [20], [21].

The DBL is a µm to cm thick region where the movement of solutes to and from metabolically active surfaces is dominated by molecular diffusion [29]. Within the DBL, biological processes, such as photosynthesis, greatly alter the chemical micro-environment [20], [21]. In some cases, DBL formation may reduce the uptake of dissolved inorganic carbon and nitrogen, and the rate of removal of O_2_ released by photosynthesis [30]–[35] but their presence is critically important to other processes such as the timing of gamete and spore release [36], [37], the deployment of extracellular enzymes [38] and maintaining steep concentration gradients of bioactive chemicals [39]. When the DBL is thick, metabolic processes can result in conditions at the organism's surface being very different to those in the overlaying bulk seawater. Using photosynthetic organisms as an example, pH and O_2_ concentrations at the organism's surface are higher than in the bulk seawater in the light due to photosynthesis, and lower in the dark due to respiration [40]–[43].

**Figure 1:**
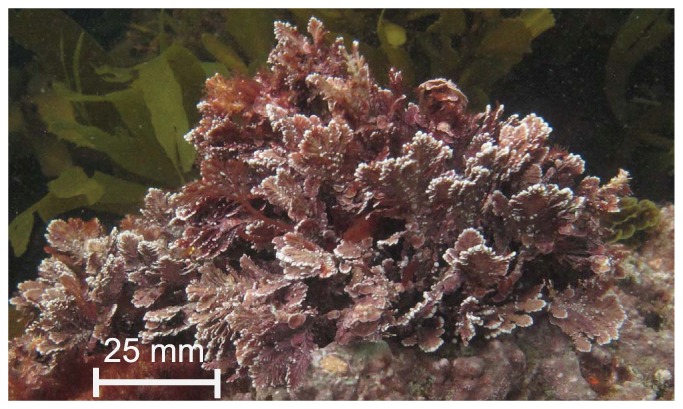
Photograph of the geniculate coralline alga *Arthrocardia corymbosa* taken at the collection site.

Our study organism, the small (<10 cm) geniculate coralline alga *Arthrocardia corymbosa* (Fig. 1), was selected as it belongs to a genus found in temperate regions worldwide [44] and has well-characterised effects on local pH (DBL scale) and seawater flow [20]. Velocities within *Arthrocardia* canopies are 80–99% slower than the bulk seawater flow, which even under fast flows results in thick (up to 18 mm) DBLs at its surface in which the pH is modified by metabolic activity [20]. It is well known that pH at the surface of calcareous species varies due to photosynthesis/respiration [20], [21], [40]. Based on this knowledge, Hurd et al (2011) hypothesised that a thick diffusion boundary layer will protect coralline algae from OA. Here we test this hypothesis by growing *A. corymbosa* in two pH treatments, one with today's seawater (ambient pH 8.05) and the second using that projected for 2100 (7.65) [3]. If the DBL acts as a protective mechanism at low pH, the greatest chance of detecting this effect would be at very slow flows were a thick DBL forms. We therefore used two velocity treatments that would promote the formation of relatively thick and thin DBLs around the coralline alga. For each pH treatment, there were two seawater velocity/DBL thickness levels, a fast flow, ‘thin DBL’ treatment with instantaneous velocities up to 5 cm s^−1^ provided by vigorous stirring in the culture tank and a slow flow, ‘thick DBL’ treatment characterised by no mechanical stirring but complete exchange of the seawater every 4.4 h to provide environmental mimicry of the periodic erosion of DBLs evident in nature [45]. Our results support the hypothesise that slow seawater flows ameliorate the negative effects of high CO_2_/low pH on coralline algae, and we suggest that habitats in which thick DBLs can form will offer a refuge for some calcareous species from OA.

## Materials and Methods

### Collection of seaweed

Thirty assemblages of the articulate coralline alga *Arthrocardia corymbosa* (Fig. 1) were collected from a *Macrocystis pyrifera*-dominated kelp forest near Karitane, South Island, New Zealand (45°38′20″S, 170°40′15″E) on 22^nd^ September 2011 using SCUBA. Field collections took place using permits provided by the New Zealand Ministry of Fisheries to the University of Otago, on publically owned land. No endangered organisms were used during this study. For a detailed description of the seaweed community composition and the light environment at the site, see Hepburn et al. [46]. Each assemblage contained 10 individual *A. corymbosa* anchored to a base of crustose coralline algae (*Mesophyllum* sp.). Assemblages were removed from the rock using hammers and chisels without touching the *A. corymbosa* individuals. Assemblages were then placed into zip-lock bags underwater and transferred to an insulated bin, in the dark, for the 30-min transport back to the laboratory. The average height of the assemblages was 5 cm. Six of these assemblages were used to assess the amount of CaCO_3_ in the seaweed tissue before the experiment began, using destructive techniques.

### Experimental design

The remaining 24 *A. corymbosa* assemblages were each placed into one of four experimental treatments for 40 days. Treatment levels were multifactorial with the two mean pH values (8.05 and 7.65 measured on the total scale) crossed with the two flow treatments (i.e. thin or thick DBL). Each combination pH/flow was replicated in 6 individual culture tanks. Assemblages were individually tied with nylon thread to circular perspex plates (60 mm diameter), that was perforated with six 1 cm diameter holes to allow seawater to flow around the seaweed and placed into one of 24 650-ml culture chambers.

pH levels within each culture chamber were maintained using a modified version of the automated culture system described by McGraw et al.[47] that uses spectrophotometric pH measurements to measure and maintain the experimental seawater pH. The culture system was housed in a walk-in growth room at 10.5°C, under a mean photon flux density (PFD) of 18 µmol photons m^−2^ s^−1^ with a 12 h:12 h light:dark cycle. Seawater (salinity  = 34 *S_A_*) was collected from Portobello Marine Laboratory, Otago Harbour, New Zealand (45°52.51′S, 170°30.9′E), filtered to 0.5 µm (Filter Pure® polypropylene spun melt) and ultraviolet sterilized (Aquastep 25 watt Ultraviolet Sterilizer). Twice a day, this water was used to fill a 150 L storage tank that fed into a mixing tank, and then into 24, independently replicated, 1 L header tanks.

Each of the 24 *A. corymbosa* assemblages was grown in an individual 650 mL Perspex flow-through culture chamber, which was attached to the outflow of a 1 L perspex header tank. Each 1 L header tank was automatically refilled from a single 1 L mixing tank. Target pH levels were achieved in each mixing tank by adding equal amounts of 0.2 M HCl and NaHCO_3_ to 1 L of seawater that was pumped from the 150 L storage tank. This method of chemical manipulation of the seawater carbonate system is chemically identical to that achieved using CO_2_ [1], [48]. Before the newly-mixed seawater was transferred to the appropriate header tank, the pH within the mixing tank was measured using the automated spectrophotometric system. If the pH was achieved within 0.03 units of the target pH, then seawater was transferred to the appropriate 1 L header tank. If the pH varied more than 0.03 pH units from the target value, the seawater in the mixing tank was sent to waste and the process was repeated until the correct pH was achieved. Using this method, the automated system delivered new seawater at the target pH to each of the 24 header tanks approximately every 4.4 h. The order in which the seawater was replenished in each of the 24 culture chambers was randomly allocated to avoid potential artefacts. Experimental treatments were also randomly allocated to header tanks to reduce potential artefacts.

Each of the 24 culture chambers were placed on a magnetic stirrer plate. A stirrer bar was placed at the bottom of each of the culture chambers to provide mechanical mixing. This was placed 5 mm below the Perspex plate to which the *A. corymbosa* assemblages were attached. In the fast flow treatment, the magnetic stirrer was set to 650 r.p.m., providing an instantaneous seawater velocity of up to 5 cm s^−1^. The seawater velocities in the culture chamber were measured using a Nortek Ventrino micro-Acoustic Doppler Velocimeter (micro-ADV). The micro-ADV was placed in the culture chamber and then the velocity measured for 120 s, at 25 Hz, with the stirrer bar set at 650 r.p.m. In the slow-flow treatment the stirrer bar was stationary and water motion was provided every 4.4 h as the seawater within each culture chamber was replenished. Therefore, although the slow-flow treatment was not stirred during the experiment, the seawater within the treatment was fully replenished more than 5 times per day, resupplying essential nutrients and removing metabolic by-products from the macroalgal surface.

The automated system controls and monitors pH in the mixing tank that feeds into each header tank. To measure the pH in each culture chamber, a Thermo Scientific Orion 720A pH/ion Meter with a Sensorex pHASE electrode was placed in the header tank on day 5, and every 5 days thereafter 4.4 hours after new seawater entered the culture tank. pH was measured on the total scale then standardized to 12°C using TRIS and 2-aminopyridine buffers. Buffers were prepared as directed by Dickson et al. [49]. TRIS buffers were standardized against a seawater buffer provided by Andrew Dickson (Scripps Institution of Oceanography). On day 1 and 40, 500 mL samples were taken to determine total alkalinity (A_T_) in the culture chambers. Samples were stored with mercuric chloride in plastic screw-top containers for later titrations. A_T_, pH on the total scale (pH_T_), salinity measurements, and temperature were used to calculate DIC. Using these values, [CO_2_], [CO_3_^2−^], and [HCO_3_^−^] were calculated using the constants of Mehrbach et al. [50] refitted by Dickson and Millero [51].

### Growth, photosynthesis and elemental analysis

The relative growth rate (RGR) of *A. corymbosa* was measured by weighing each replicate assemblage at the beginning of the experiment and again after 40 days. RGR was calculated using the following formula [52]:
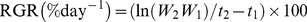
(1)where *t*_1_ = day 1, *t*_2_ = day 40, *W*_1_ is wet weight on day 1 and *W*_2_ is wet weight on day 40.

*A. corymbosa* reproduced during the experiment. Recruitment of juveniles was measured on the perspex plates to which the seaweed assemblages were attached. The plates were photographed using a Cannon Powershot A620 digital camera attached to a Zeiss Axiostar Plus microscope at 20 times magnification. For each perspex plate, the number of individual recruits was recorded on a random 10×10 mm sub-sample, using the software ImageJ 1.42q [53]. The mean surface area of the recruits for each replicate was estimated from 10 randomly-selected recruits from each plate.

The *F*_v_ */F*_m_ of one individual from each replicate assemblage was measured at the end of the experiment, using a Pulse Amplitude Modulated (PAM) chlorophyll fluorescence meter (Diving PAM, Walz, Germany). This model has a red light-emitting diode, and both gain and dampening were set to 2. On all occasions *F*_o_ was >130 before measurements were made. Each assemblage was dark-adapted for 30 min prior to measurements [54]. Relative electron transport rates (r*ETR*_max_) for each replicate assemblage were determined using the rapid light curve function. *A. corymbosa* individuals were exposed to 9 light steps of 20 s duration with the irradiance increasing from 0 to 1234 µmol photons m^−2^ s^−1^. Equations using least-squares non-linear regression were then used to determine r*ETR*_max_, *α* (light-use efficiency), *β* (photoinhibition) and *I*_k_ (light saturation point) [55]. Pigment content was determined on day 40 using the pigment extraction methods from Sampath-Wiley & Neefus [56] for phycobillins (phycocyanin and phycoerythrin) and Richie [57] using ethanol for chlorophyll *a*.

%C, %N, δ^13^C and δ^15^N were analysed in the organic tissue of the seaweeds on day 40 by removing all inorganic tissue in 1 M HCl, washing with purified fresh water, then drying at 80°C and grinding samples in a porcelain mortar and pestle. Sub-samples of 1.5 mg were then combusted in a CE NA1500 Elemental Analyzer (Carlo-Erba instruments) interfaced to a Europa Scientific 20–20 update continuous flow mass spectrometer. Corrections for drift were made automatically every 5 samples from a standard (EDTA) with a known isotope ratio. C:N ratios (molar ratio) were calculated.

To determine the % magnesium calcite (MgCO_3_) on day 40, dried seaweed samples were bleached to remove organic tissue then ground to a fine powder then examined under X-ray diffractometry (XRD) for carbonate mineralogy using the methods described by Smith et al.[58] and calibration equations from Gray and Smith [59].

The difference in pH between the mixing and culture chambers measured on day 1 and every 5 days thereafter (see Experimental Design) were used to see if metabolic activity due to the uptake and release of CO_2_ during photosynthesis or respiration [60], [61] differed with treatment. pH measurements were first converted into H^+^ concentrations [H^+^] (H^+^ = 10^−pH^) and the change in [H^+^] determined by deducting [H^+^] in the culture chamber by the concentration in the mixing chamber.

### Diffusion boundary layer thickness and pH conditions at seaweed surface

The thickness of the diffusion boundary layer (DBL) around each *Arthrocardia corymbosa* assemblage was measured in each treatment under light and dark conditions on day 1 and 40, using a Presens 50 *µ*m oxygen microoptode attached to a World Precision Instruments Inc M3301R manual micro-manipulator. The microoptode was placed at the base of each seaweed assemblage and measurements were made for one minute at set distances (0.1 mm for the first mm, then 1 at mm intervals thereafter) from 0 to 40 mm above the base above [20]. This method was used instead of measuring from the tip of the thallus upwards, because most the biomass resides below the tip and thallus movement at the tip under fast flows broke the electrode.

pH was estimated at the seaweed surface using the O_2_ microoptode measurements and results of a previous experiment using *A. corymbosa* at pH 8.00 and 7.65 [20]. These results demonstrated a strong relationship between H^+^ and O_2_ concentrations within the DBL (R^2^ = 0.82; due to the uptake and release of CO_2_ and O_2_ during photosynthesis and respiration) and showed that the DBL thickness measured using changes in H^+^ and O_2_ are of equal size. O_2_ concentration can therefore be confidently used as a proxy for [H^+^]. We first converted O_2_ concentration to [H^+^] using separate relationships in the dark and in the light at both pH 7.65 and pH 8.05. Then, the calculated [H^+^] deviations at the surface of *A. corymbosa* were multiplied by deviations in the bulk seawater measured during the current study (see above section). Finally, [H^+^] was used to calculate Ω_C_ based on total alkalinity measurements made in the bulk seawater.

### Estimation of net calcification

The net calcification rate of one *A. corymbosa* individual from each replicate assemblage was estimated from the amount of inorganic tissue gained over 40 days relative to the starting weight of inorganic tissue measured in individuals sacrificed at day 0. Formula (1) for RGR was used to calculate net calcification, except the coefficient *W* was replaced with *WC* (the weight of CaCO_3_. *WC* is determined by the following formula:
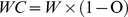
(2)where *W* = g dry weight and *O* =  the proportion of organic tissue (g^−1^ of dry weight). For *WC*_1_, *O* of the initial sacrificed *A. corymbosa* sample is used, all other parameters were obtained from the experimental *A. corymbosa* on day 0 and 40.

The proportion of organic tissue (*O*) was determined by taking one whole *A. corymbosa* thallus from each replicate (0.06±0.01 g of dried seaweed), then dissolving the sample in 10% HCl and re-weighing it. The ratio of wet:dry weight for day 0 replicates was calculated by measuring the wet weight of 20 *A. corymbosa* samples collected from the field at day 0, then dividing by their weight after drying at 80°C for 24 h. This ratio had an R^2^ of 0.94.

Ω (saturation states of minerals) has been used in many past studies to show the relationship between net calcification and carbonate chemistry where, as Ω declines, net calcification also declines for many organisms [62]. Ω_C_ (saturation state for calcite), which is proportional to [CO_3_^2−^] in the seawater, may have no direct physiological role in controlling net calcification [63], although related carbonate parameters (namely H^+^, HCO_3_^−^ and Ca^2+^) likely do. We refer to Ω_C_ throughout the text but do not infer that it controls any aspect of calcification directly.

### Statistical analyses

RGR, net calcification rates, recruit numbers and sizes, all pigments, the % MgCO_3_, % CaCO_3_, C:N, δ^13^C, δ^15^N, r*ETR*_max_, *α*, *β*, I_k_, and H^+^ deviations in the bulk seawater and H^+^ concentrations at the surface were analysed using an analysis of variance (ANOVA) with the pH and the level of seawater flow/DBL thickness used as the two fixed factors, with the interaction term also included in the model. *F*_v_/*F*_m_ was analysed as a repeated-measures type analysis of variance (ANOVAR), with time as the random factor and pH and flow/DBL thickness treatment the fixed factors. All were tested for homoscedasity and normality prior to analysis. Recruit number data failed these assumptions and were log (*X*+1) transformed. When *p*-values <0.05 were detected within the main effects, Tukey-Honestly Significant Difference (hereafter THSD) post-hoc tests were used to determine differences between treatment levels. All statistical analyses were performed in R version 2.7.0 [64].

## Results

**Figure 2:**
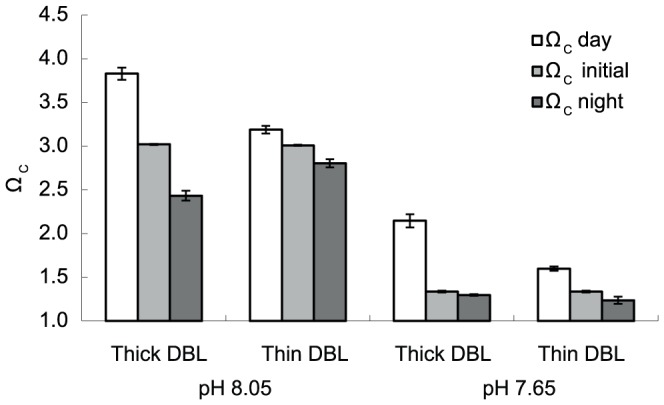
Carbonate saturation state (Ω_C_) of seawater at the surface of the coralline alga *Arthrocardia corymbosa*. Computed values of the carbonate saturation state (Ω_C_) of seawater at the surface of the coralline alga *Arthrocardia corymbosa*, in the light and dark, under four combinations of pH and DBL (diffusion boundary layer) thickness (see Supplementary Information). The Ω_C_ initial value is that of new seawater that has been introduced into the culture chamber. ‘Ω_C_ day’ is the calculated Ω_C_ at the seaweed surface after 4.4 h incubation in the light, and Ω_C_ night is that after 4.4 h in the dark. The bars represent the mean ± standard error of 6 individual replicates, and in some the error bars are too small to be seen.

The DBL in the fast flow treatment was thinner than those in the slow flow treatments (mean 0.03 and 14.77 mm respectively, see Table S1). The calcite saturation state at the surface of the seaweed at pH 7.65 was very different for the thin compared to thick DBL treatment. Ω_C_ at the surface of the seaweed was estimated at the start (initial) and end of the 4.4 h cycle in both the light and dark (Fig. 2). At pH 8.05, Ω_C_ at the surface of the seaweed increased in the light and decreased in the dark (relative to the initial value), although the range in Ω_C_ was less in the thin compared to the thick DBL treatment (Fig. 2, Table S1). For the pH 7.65/thick DBL treatment, Ω_C_ was also higher in the light, but at night Ω_C_ was comparable to the initial Ω_C_. In the pH 7.65/thin DBL treatment, Ω_C_ in the dark also remained similar to the initial value, however Ω_C_ in the light was significantly lower than in any other treatment (Table S2, *P*<0.01; Fig. 2). Therefore, in the pH 7.65/thin DBL treatment, the seaweeds encounter a lower mean value of Ω_C_ over the day/night cycle compared to the thick DBL/pH 7.65 treatment.

**Figure 3:**
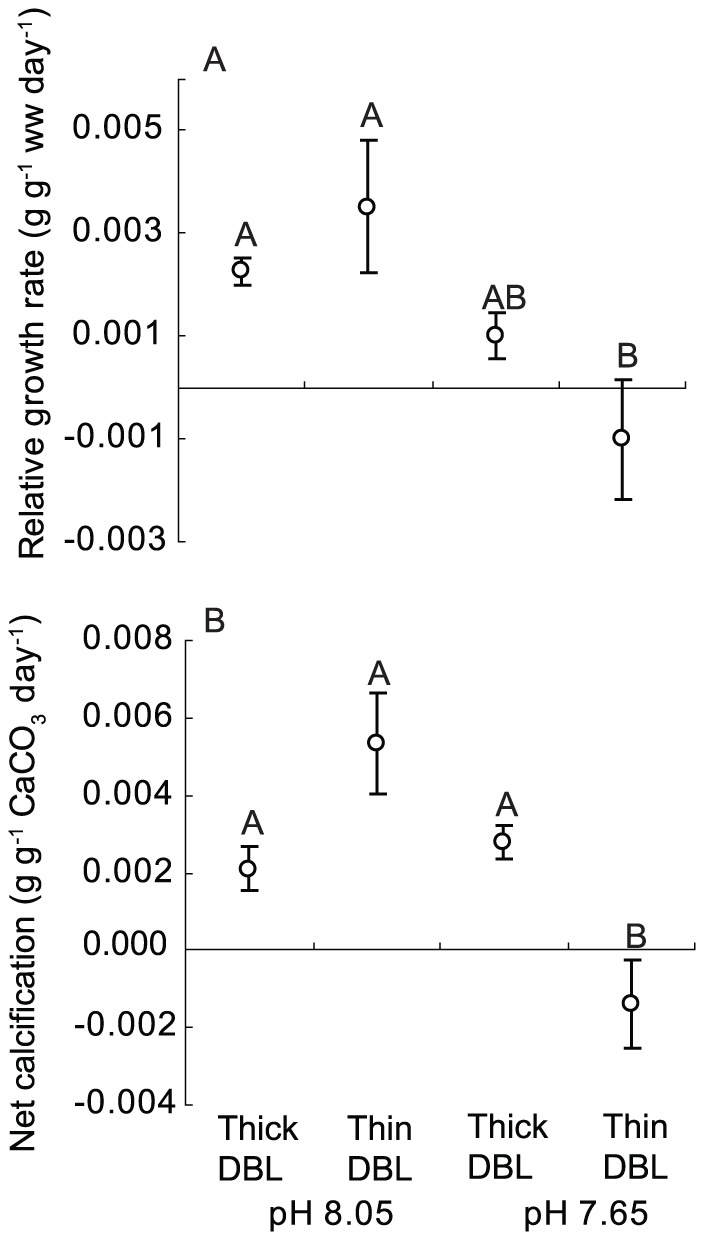
Effect of DBL thickness and pH on the relative growth rate and net calcification rate. The effect of DBL thickness/seawater flow and pH on the A) relative growth rate and B) net calcification rate of the coralline alga *Arthrocardia corymbosa*. Thick DBL (diffusion boundary layer)  =  no mechanical stirring with regular replenishment of the seawater media and fast flow  = >5 cm s^−1^. The bars represent the mean ± standard error of 6 individual replicates.

There was a significant effect of pH treatment on growth rates (*P* = 0.01), with higher growth at pH 8.05 than 7.65, no significant effect of flow/DBL thickness (*P* = 0.67) but and a near-significant interaction term (*P* = 0.08 Table S2). Trends in net calcification mirrored those for growth rates (Fig. 1b), with negative rates of net calcification observed in the thin DBL/pH 7.65 treatment. The only difference between the two diagnostics being that the interaction between DBL thickness and pH treatments was significant for net calcification (*P*<0.01). The significant interaction term was due differences in the effect of DBL thickness on calcification at pH 8.05 and 7.65. This significant interaction term was due to the effect of DBL thickness being opposite at pH 8.05 compared to 7.65, where the mean calcification rates were highest in thin DBL treatments at pH 8.05 and lowest in thin DBL treatments at pH 7.65 (Fig. 3a). The magnitude of the change in rates of growth (Fig. 3a) and calcification (Fig. 3b) across the treatments were relatively comparable.

**Figure 4:**
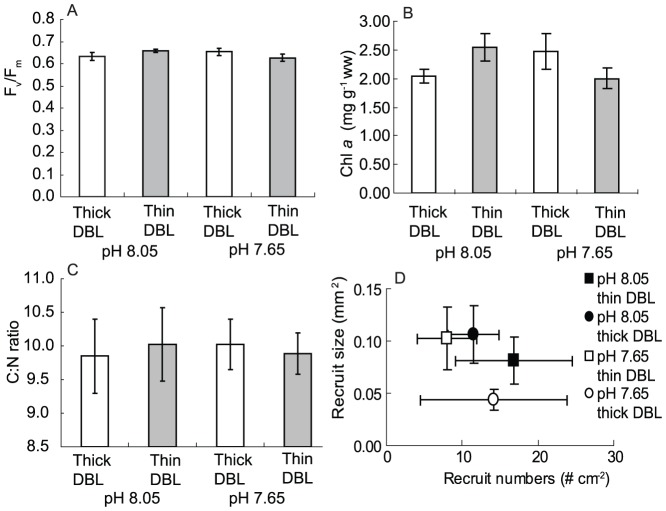
Diagnostics of the health of the coralline alga *Arthrocardia corymbosa*. Physiological and reproductive metrics used as diagnostics of the health of the coralline alga *Arthrocardia corymbosa* grown under four combinations of DBL (diffusion boundary layer) thickness and pH. (a) photosynthetic competence (F_V_:F_M_), (b) chlorophyll *a* content, (c) elemental stoichiometry (C:N molar ratio) and (d) reproductive output (number and size of recruits). The bars and points represent the mean ± standard error of 6 individual replicates.

Although there were clear effects of pH and DBL thickness on rates of growth and net calcification (Fig. 3), little change was observed for other diagnostics of seaweed health. No significant differences between treatments were detected for photosynthetic competence (F_V_:F_M_), chlorophyll *a* content and the C:N ratio (Fig. 4a–c; *P*>0.3 on all occasions). Similarly, other measures of seaweed health that included accessory pigment composition, photosynthetic capacity and elemental composition did not differ among treatment (*P*>0.21 on all occasions, Tables S2–S3, Figures S1–S4). The seaweeds reproduced during the 40 d experiment, and the number and size of the recruits were also comparable for all treatments (*P*>0.3 on all occasions, Table S2, Fig. 4d).

## Discussion

Our study revealed contrasting findings - differences in the rates of growth and net calcification between treatments, yet no evidence from the other metrics that physiological or reproductive performance was altered by pH and/or water motion/DBL thickness. The most compelling explanation to reconcile these seemingly disparate findings is that the regular exposure to lower pH in the thin DBL/pH 7.65 treatment resulted in a lower mean value of Ω_C_ at the seaweed surface hence increasing the chemically-induced dissolution of calcium carbonate structures at the surface of the organism, rather than reducing biologically-mediated changes to gross calcification (as net growth of adults was negative in this treatment). Importantly, our results support our initial hypotheses, and those of Hurd et al. [21] who suggested that OA should have a lesser effect on the calcification rates of photosynthetic species growing in slow-flows than those in fast-flows. The present study confirms that in slow-flow conditions, where the DBL is thick, carbonate chemistry at the seaweed surface is conducive to higher net calcification (hence influencing non-significant trends in growth, as the two are intertwined in coralline algae [65]). This occurs through a combination of metabolic processes and boundary-layer dynamics. Conversely, in fast-flows the calcifying seaweed surface is constantly in contact with low pH seawater, due to erosion of the DBL, most likely leading to increased dissolution.

**Figure 5:**
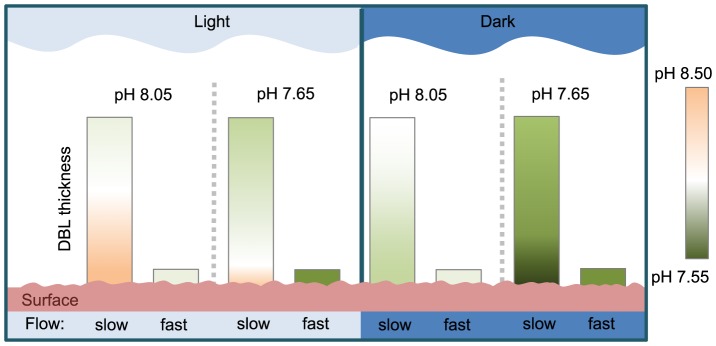
Modification of seawater pH at the surface of the coralline algae. Conceptual scheme of the interplay between seawater carbonate chemistry (i.e. pH), organismal modification of the local chemical environment during a day/night cycle and flow regime illustrating the potential feedbacks between these inter-related processes. Slow flow denotes the slow flow conditions used in our study.

The interplay of bulk seawater pH, organismal modification of pH, and water motion are summarised in Fig. 5. These factors interact to set up a pronounced diurnal cycle of vertical pH gradients from the calcified surface of the organism to the outermost extent of the DBL. Thus, an ecomechanical [28] view of environmental heterogeneity reveals that coralline algae can encounter a wide range of conditions with respect to diurnal changes in pH from almost constant conditions in fast flows, to a wide range of pH and Ω_C_ over the diurnal cycle in slow flows. Slow flows will likely provide periods of respite from constantly low pH (or Ω_C_) in a future ocean influenced by OA, allowing increased net calcification. Such slow-flow conditions may act as a stop-gap, enabling calcifiers to adapt or acclimate to lower pH conditions projected for the coming decades [21].

Fast flow rates did not significantly increase the growth or photosynthesis of *Arthrocardia corymbosa* here, nor was there an effect of flow or pH on photosynthetic physiology. There are three possible reasons why fast flow did not enhance growth at ambient pH. First, the high variability in seaweed physiological responses here masked any significant trends. If we had used higher numbers of culture tanks here (*n*>6), it may have resulted in significant differences as a result of increased power during the analysis. Second, *A. corymbosa's* photosynthetic rates were saturated for CO_2_ at the irradiance levels used here at ambient pH. This suggestion is supported by the observation that the δ^13^C data indicate no change in the proportion of diffusive CO_2_ used by *A. corymbosa* grown under fast and slow flows [66]. A third explanation is that the replenishment of the DBL every 4.4 hours provided sufficient inorganic nitrogen to *A. corymbosa*. While the DBL was thick enough to reduce the dissolution of calcareous structures in the pH 7.65/thick DBL treatment, its formation did not result in inorganic nitrogen or DIC limitation because the organic carbon and nitrogen content (C:N ratio) of *A. corymbosa* did not vary significantly. This is in line with recent findings that the DBL only acts to limit inorganic nitrogen or carbon under conditions where the concentration in the bulk seawater is already relatively low for some species [67], [68].

Our suggestion that the DBL acts to protect coralline algae from OA is in line with a growing body of evidence that DBLs can be beneficial to seaweeds. For example, seaweeds sense seawater flow via changes in the carbonate system within the DBL and thereby control the timing of gamete and spore release [36], [37]; extracellular enzymes are deployed at the seaweed surface within the DBL [38] and they help maintain steep concentration gradients of anti-fouling chemicals [39]. Although they can limit nutrient uptake in some circumstances, velocities required for maximal nutrient uptake are dependent on seaweed morphology, and are usually between 0.5 and 2 cm s^−1^ [35], [69]–[71]. The effects of OA *in situ* for calcareous photosynthetic species, and the epibionts that grow on their surface, will likely be directly related to the micro-pH environment encountered by individual organisms [21], [72], [73].

The extent of the impacts of OA for some marine organisms will therefore be dependent on their locality and factors involved in modifying the DBL thickness. Species with complex morphologies that trap or slow flow around them will have thicker DBLs [20], [21], [74], potentially resulting in more robust responses to OA. Likewise, species with faster metabolic rates, or with semi-enclosed spaces where calcification (or DIC uptake for calcification) and photosynthesis co-occurs, will encounter greater biological modification of pH at the site of calcification [75]–[77], also resulting in more robust responses to the effects of OA compared to species with slow metabolic rates and/or external calcification sites [78]. Importantly, organisms in areas with high wave energy or fast currents will likely show the typical species-specific responses to OA (usually in the form of reductions in net calcification rates and, subsequently, in growth rates and the survival of organisms) [79]. In contrast, the impacts of OA may be reduced for organisms living within areas of slow flow, such as in embayments, wide estuaries or within the canopies of larger seaweed that can reduce flow rates [34], [80], [81] and alter pH conditions [23], [82], [83]. Therefore, the positive effect of a thick DBL shown here at pH 7.65 indicates that the prevailing view that the DBL negatively impacts primary producers needs to be re-evaluated [34], [84].

There were no statistical differences in the size or numbers of recruits after 40 days in our experiment. This may mean that either 1) different processes control the growth and development of micro-stages of calcareous seaweed, as with non-calcareous seaweed and coral species [85]–[87], or that 2) the growth and net calcification of micro-stage coralline algae is very slow, meaning that if the experiment was longer then the full effects of OA and flow may have been observed on recruit sizes. The growth rates of juvenile *A. corymbosa* in a previous experiment did not resemble to those of the adults for the first 40 days [82], and only after 22 weeks did they mirror those of the adults (M.Y. Roleda et al., *in prep*). Because recruits in specific culture tanks may have germinated at different times, it is possible that after a longer duration they may have mirrored the adult growth and net calcification rates. Recruit numbers were also unaffected by pH and flow, though there was a non-significant tendency for recruit numbers to be higher in treatments with faster adult net calcification rates, suggesting a trade-off between energetic demands induced by faster dissolution rates and the energetic demands of reproduction. However, the fact that recruit sizes and numbers were not significantly altered by OA confirms previous research [82] that indicates that the effects of OA on coralline recruitment may be species specific.

In addition to the physiological insights gained from this study, our work has important implications on how OA culture experiments are conducted. Flow conditions within experiments are rarely detailed, making it very difficult to predict the carbonate chemistry at the surface of organisms, which therefore makes comparisons between studies difficult. Vigorous water motion via mixing (bubbling or stirring or shaking) is often recommended [48], [88] to reduce the formation of diffusion boundary layers around marine primary producers, thereby maximizing nutrient uptake; however this mixing will exacerbate the negative effects of OA as the protective diffusion boundary layers will not form. Conversely, conducting experiments with no/slow-flow will result in markedly different carbonate chemistry conditions at the surface of the cultured organism, which will depend largely on the metabolic activity of the organism [21]. Therefore, if experiments are conducted on the same study species but under different flow regimes, the biological responses could vary, making it difficult to compare, and extrapolate, the responses of organisms to OA. We provide the first examination of how the DBL could influence the growth of calcareous species under conditions simulating OA. However, the source of this flow used here does not exactly mimic the source of flow in the field. Vortex flow here was produced from beneath the seaweed, whereas in the field flow will usually either be unidirectional or oscillatory. Long-term measurements of flow at a scale relevant to understorey species is needed before conclusions can be drawn about the role of the DBL in situ. That withstanding, we recommend that seawater velocity is monitored and reported for all OA research, along with the other important environmental factors (e.g. photon flux density, photoperiod, nutrients, rate of seawater exchange), in order to better facilitate the interpretation of results.

Seaweed beds could provide regions of slow flow/higher pH where calcifying organisms could subside in future low pH seawater. The physical and chemical environment within a seaweed canopy is very different to that of the bulk seawater – i.e., the seawater flow is much reduced [89] and the metabolism of seaweed also results in higher pH values within areas influenced by their photosynthesis during the day [90], [91]. However, further research is required to fully examine the role of kelp canopies on understorey organisms. For example, the flow rates in our slow flow treatment were zero for over four hours, and while velocity can reach zero within seaweed canopies [89], the temporal dynamics will be a function of seaweed density, size and bulk seawater velocity, which is usually greater than zero [20], [89], [92], [93]. Future research should examine what protective role the DBL could play at a greater range of seawater velocities, and for a greater range of species, than those used here. In addition, kelp canopies alter other environmentally important variables, such as light intensity [14], [94], [95] or nutrient concentrations [96], which could interact with OA or other environmental stressors to influence calcification, photosynthetic or growth rates of coralline algae [97], [98].

The experimental findings, in conjunction with previously published flow measurements beneath seaweed canopies [20], [89], provide the first insights into how the localised chemical and hydrodynamic processes that occur at the surface of the understorey species could interact with global-scale processes such as ocean acidification. Together, they reveal that regions of slow flow may help to conserve important biodiversity of calcifiers in selected regions. Rau et al. [99] discuss the need for novel conservation strategies to offset the effects of OA, and put forward several options including boosting the resilience of calcifiers to OA through breeding programmes. Rau et al. [99] also advocated the potential management of local carbonate chemistry using geochemical modification of seawater, but pointed out that such an approach would be time-consuming, and expensive even to modify a hundreds of m^2^ of the seafloor/reefs. If our results are transferable to the field, we propose that such temporal and logistical hurdles could be overcome by maintaining seaweed beds, characterised by localised biological modification of pH and seawater flow, as potential refugia (km^2^) from OA.

## Supporting Information

Figure S1 **The proportion of total organic tissue, CaCO_3_ and MgCO_3_ in** ***A. corymbosa*** **.** The proportion of total organic tissue, CaCO_3_ and MgCO_3_ in *A. corymbosa* after 40 days in thick and thin DBL treatments at pH 7.65 and 8.05.
(EPS)

Figures S2 **Chlorophyll fluorescence parameters of** ***A. corymbosa*** **.** (a) Relative maximum electron transport (r*ETR*_max_), (b) degree of photoinhibition (β), (c) light use efficiency (α) and (d) irradiance saturation point (I_K_) of *A. corymbosa* measured using chlorophyll fluorescence at day 40 in each treatment.
(EPS)

Figure S3 **Pigment content of** ***A. corymbosa*** **.** (A) phycoerythrin and (B) phycocyanin content of *A. corymbosa* grown for 40 days with thick and thin DBLs at pH 7.65 and 8.05.
(EPS)

Figure S4 **δ^13^C and δ^15^N of** ***A. corymbosa*** **.** Organic tissue δ^13^C and δ^15^N of *A. corymbosa* grown for 40 days with thick and thin DBLs at pH 7.65 and 8.05.
(EPS)

Table S1 **Physical conditions during the experiment and the seawater carbonate chemistry parameters.**
(DOCX)

Table S2 **Analysis of variance results of biotic (non-elemental and pigment) responses of** ***A. corymbosa*** **to the experimental treatments.**
(DOCX)

Table S3 **Analysis of variance results of elemental and pigment biotic responses of** ***A. corymbosa*** **to the experimental treatments.**
(DOCX)
