# Correction: Diffusion Boundary Layers Ameliorate the Negative Effects of Ocean Acidification on the Temperate Coralline Macroalga *Arthrocardia corymbosa*

**DOI:** 10.1371/journal.pone.0109468

**Published:** 2014-09-26

**Authors:** The PLOS ONE Staff

There are errors in the author affiliations. The affiliations should appear as shown here:

Christopher E. Cornwall^1^, Philip W. Boyd^2^, Christina M. McGraw^3,4^, Christopher D. Hepburn^7^, Conrad A. Pilditch^5^, Jaz N. Morris^1^, Abigail M. Smith^7^, Catriona L. Hurd^1,6^

1 Department of Botany, University of Otago, Dunedin, New Zealand, 2 National Institute for Water and Atmospheric research (NIWA) Centre of Physical and Chemical Oceanography, Dunedin, New Zealand, 3 School of Chemistry and Biochemistry, Clark University, Worcester, Massachusetts, United States of America, 4 School of Science and Technology, University of New England, Armidale, Australia, 5 Department of Biological Sciences, University of Waikato, Hamilton, New Zealand, 6 Institute for Marine and Antarctic Studies, University of Tasmania, Hobart, Tasmania, Australia, 7 Department of Marine Sciences, University of Otago, Dunedin, New Zealand
