# Supplementary material for: Diffusion Boundary Layers Ameliorate the Negative Effects of Ocean Acidification on the Temperate Coralline Macroalga Arthrocardia corymbosa
Source: PLoS One. 2014 May 13;9(5):e97235. doi: 10.1371/journal.pone.0097235 (PMC4019523; doi:10.1371/journal.pone.0097235)
Supplement: Table S1 — Physical conditions during the experiment and the seawater carbonate chemistry parameters. (DOCX) [file pone.0097235.s005.docx]

**Table S1**. Physical conditions during the experiment and the seawater carbonate chemistry parameters.

| Parameter | pH 8.05 Fast flow | pH 8.05 Slow flow | pH 7.65 Fast flow | pH 7.65 Slow flow |
| --- | --- | --- | --- | --- |
| pH_T_ | 8.04 (0.01) | 8.05 (0.01) | 7.65 (0.01) | 7.65 (0.01) |
| Temperature (°C) | 10.53 (0.01) | 10.53 (0.01) | 10.53 (0.01) | 10.53 (0.01) |
| DIC (*μ*mol kg^-1^) | 1973 (4) | 1955 (15) | 2071 (12) | 2031 (12) |
| A_T_ (*μ*mol kg^-1^) | 2152 (3) | 2140 (16) | 2114 (11) | 2074 (12) |
| *p*CO_2_ (*μ*atm) | 372 (2) | 368 (3) | 998 (13) | 966 (1) |
| HCO_3_^-^ (*μ*mol kg^-1^) | 1830 (9) | 1813 (14) | 1973 (11) | 1934 (11) |
| CO_3_^2-^ (*μ*mol kg^-1^) | 127 (0.1) | 126 (1.1) | 55 (0.3) | 55 (0.6) |
| CO_2_ (*μ*mol kg^-1^) | 16 (0.1) | 16 (0.1) | 43 (0.6) | 42 (0.1) |
| DBL thickness (mm) | 0.03 (0.01) | 14.53 (2.61) | 0.02 (0.01) | 15.00 (2.03) |
