# Supplementary material for: Diffusion Boundary Layers Ameliorate the Negative Effects of Ocean Acidification on the Temperate Coralline Macroalga Arthrocardia corymbosa
Source: PLoS One. 2014 May 13;9(5):e97235. doi: 10.1371/journal.pone.0097235 (PMC4019523; doi:10.1371/journal.pone.0097235)
Supplement: Table S2 — Analysis of variance results of biotic (non-elemental and pigment) responses of A. corymbosa to the experimental treatments. (DOCX) [file pone.0097235.s006.docx]

**Table S2**. Analysis of variance results of biotic (non-elemental and pigment) responses of *A. corymbosa* to the experimental treatments. *p* values 0.05 are in bold.

| Parameter | Factor | Degrees of Freedom | *F* value | *p* value |
| --- | --- | --- | --- | --- |
| RGR | pH | 1 | 10.14 | **< 0.01** |
|  | Flow | 1 | 0.18 | 0.67 |
|  | pH × Flow | 1 | 3.25 | 0.08 |
|  | Residuals | 20 |  |  |
| Net calcification | pH | 1 | 10.11 | **< 0.01** |
|  | Flow | 1 | 0.27 | 0.61 |
|  | pH: Flow | 1 | 15.35 | **< 0.01** |
|  | Residuals | 20 |  |  |
| Recruit numbers | Flow | 1 | < 0.01 | 0.95 |
|  | pH × Flow | 1 | 0.64 | 0.44 |
|  | Residuals | 20 |  |  |
| Recruit size | pH | 1 | 1.12 | 0.30 |
|  | Flow | 1 | 0.47 | 0.50 |
|  | pH × Flow | 1 | 3.13 | 0.09 |
|  | Residuals | 20 |  |  |
| *F*_v_ / *F*_m_ | pH | 1 | 1.22 | 0.28 |
|  | Flow | 1 | 0.41 | 0.53 |
|  | pH × Flow | 1 | 0.35 | 0.56 |
|  | Time | 1 | 156.72 | **< 0.01** |
|  | Residuals | 42 |  |  |
| r*ETR*_max_ | pH | 1 | 0.05 | 0.83 |
|  | Flow | 1 | 1.43 | 0.25 |
|  | pH × Flow | 1 | 2.50 | 0.14 |
|  | Residuals | 19 |  |  |
| *α* | pH | 1 | 0.71 | 0.41 |
|  | Flow | 1 | 1.39 | 0.25 |
|  | pH × Flow | 1 | 0.02 | 0.90 |
|  | Residuals | 19 |  |  |
| *β* | pH | 1 | 0.01 | 0.97 |
|  | Flow | 1 | 4.05 | 0.06 |
|  | pH × Flow | 1 | 0.41 | 0.53 |
|  | Residuals | 19 |  |  |
| I_K_ | pH | 1 | 0.03 | 0.87 |
|  | Flow | 1 | 2.45 | 0.13 |
|  | pH × Flow | 1 | 1.26 | 0.28 |
|  | Residuals | 19 |  |  |
| Δ[H^+^] night | pH | 1 | 1.09 | 0.30 |
|  | Flow | 1 | 8.43 | **<0.01** |
|  | pH × Flow | 1 | 3.86 | 0.06 |
|  | Residuals | 108 |  |  |
| Δ[H^+^] day | pH | 1 | 100.62 | **<0.01** |
|  | Flow | 1 | 1.31 | 0.26 |
|  | pH × Flow | 1 | 0.57 | 0.46 |
|  | Residuals | 142 |  |  |
| Surface[H^+^] day | pH | 1 | 835.30 | **<0.01** |
|  | Flow | 1 | 109.91 | **<0.01** |
|  | pH × Flow | 1 | 0.65 | 0.43 |
|  | Residuals | 20 |  |  |
| Surface[H^+^] night | pH | 1 | 1030.87 | **<0.01** |
|  | Flow | 1 | 13.67 | **<0.01** |
|  | pH × Flow | 1 | 26.30 | **<0.01** |
|  | Residuals | 20 |  |  |
