# Supplementary material for: Diffusion Boundary Layers Ameliorate the Negative Effects of Ocean Acidification on the Temperate Coralline Macroalga Arthrocardia corymbosa
Source: PLoS One. 2014 May 13;9(5):e97235. doi: 10.1371/journal.pone.0097235 (PMC4019523; doi:10.1371/journal.pone.0097235)
Supplement: Table S3 — Analysis of variance results of elemental and pigment biotic responses of A. corymbosa to the experimental treatments. (DOCX) [file pone.0097235.s007.docx]

**Table S3.** Analysis of variance results of elemental and pigment biotic responses of *A. corymbosa* to the experimental treatments.

| Parameter | Factor | Degrees of Freedom | *F* value | *p* value |
| --- | --- | --- | --- | --- |
| Chl *a* | pH | 1 | 0.19 | 0.67 |
|  | Flow | 1 | 0.01 | 0.96 |
|  | pH × Flow | 1 | 2.97 | 0.10 |
|  | Residuals | 20 |  |  |
| Phycocyanin | pH | 1 | 0.28 | 0.60 |
|  | Flow | 1 | 1.69 | 0.21 |
|  | pH × Flow | 1 | 0.39 | 0.54 |
|  | Residuals | 20 |  |  |
| Phycoerythrin | pH | 1 | 0.48 | 0.50 |
|  | Flow | 1 | 0.89 | 0.36 |
|  | pH × Flow | 1 | 0.68 | 0.42 |
|  | Residuals | 20 |  |  |
|  |  |  |  |  |
| δ^13^C | pH | 1 | 0.05 | 0.83 |
|  | Flow | 1 | 0.52 | 0.48 |
|  | pH × Flow | 1 | 2.02 | 0.17 |
|  | Residuals | 20 |  |  |
| δ^15^N | pH | 1 | 0.45 | 0.51 |
|  | Flow | 1 | 0.27 | 0.61 |
|  | pH × Flow | 1 | 0.10 | 0.76 |
|  | Residuals | 20 |  |  |
| C:N | pH | 1 | 0.22 | 0.64 |
|  | Flow | 1 | 0.01 | 0.99 |
|  | pH × Flow | 1 | 0.02 | 0.88 |
|  | Residuals | 18 |  |  |
| % MgCO_3_ | pH | 1 | 0.06 | 0.81 |
|  | Flow | 1 | 0.03 | 0.87 |
|  | pH × Flow | 1 | 0.09 | 0.77 |
|  | Residuals | 20 |  |  |
